# Association between the use of colistin for short-term treatment of Gram-negative bacterial infections and the emergence of colistin-resistant Enterobacteriaceae in swine from selected swine farms in Thailand

**DOI:** 10.1371/journal.pone.0238939

**Published:** 2020-10-05

**Authors:** Pariwat Poolperm, Teerawit Tangkoskul, Chakkrapong Seenama, Naruemon Maknakhon, Visanu Thamlikitkul

**Affiliations:** 1 Faculty of Veterinary Medicine, Kasetsart University, Nakhon Pathom, Thailand; 2 Division of Infectious Diseases and Tropical Medicine, Department of Medicine, Faculty of Medicine Siriraj Hospital, Mahidol University, Bangkok, Thailand; University of Georgia, UNITED STATES

## Abstract

Long-term use of colistin for preventing Gram-negative bacterial infections in food animals was prohibited in Thailand in 2017, but it is permitted for short-term treatment. This study aimed to investigate association between the use of colistin for short-term treatment of infection and the emergence of colistin-resistant Enterobacteriaceae in swine. The current study was conducted at 2 selected swine farms in Thailand. Neither farm has used colistin to prevent infection for longer than 1 year. Rectal swabs were collected from the same 66 pigs at birth, and on days 7, 14, 21, 28, and 60. Colistin was used to treat sick pigs for up to 3 days. Additional rectal swabs were collected during colistin treatment. Rectal swabs were analyzed for colistin-resistant Enterobacteriaceae and the *mcr-1* gene. Results revealed that colistin-resistant Enterobacteriaceae were absent at birth. Some pigs at both farms had diarrhea and received colistin treatment during days 2–27. Colistin-resistant Enterobacteriaceae were detected in 13.3–50.0% of sick and healthy pigs. No sick pigs were observed during days 28–60, and colistin was not used during that period. Colistin-resistant Enterobacteriaceae were detected in 2.8–10.0% of healthy pigs on day 28, and in 0–3.4% of healthy pigs on day 60. The *mcr-1* gene was detected in 57.6% of colistin-resistant Enterobacteriaceae isolates. Short-term treatment with colistin was found to be associated with the emergence of colistin-resistant Enterobacteriaceae in swine. Colistin-resistant Enterobacteriaceae rapidly emerged after colistin use, and rapidly decreased or disappeared after its discontinuation.

## Introduction

Colistin has been one of the last-resort antibiotics for treatment of human infection caused by carbapenem*-*resistant *Acinetobacter baumannii*, carbapenem*-*resistant *Pseudomonas aeruginosa*, and carbapenem*-*resistant Enterobacteriaceae (CRE) over the past decade [1]. Colistin has also been widely used in livestock for the prevention, control, and treatment of Gram-negative bacterial infections [2, 3]. Use of colistin in food animals and human beings, its association with colistin resistance, and/or increased colistin minimum inhibitory concentration (MIC) in Gram-negative bacteria in human beings and animals has been reported from many countries [4–16]. Mechanisms of colistin resistance are usually associated with chromosome-mediated mutations [17]. However, the mobilized, plasmid-borne, colistin resistance gene (*mcr-1*) was found in *Escherichia coli* isolated from pigs and human beings in 2015 [18]. Since that discovery, *mcr-1*-mediated colistin-resistant Enterobacteriaceae have been detected in animals, healthy individuals, and patients in many countries around the world [19]. The emergence of the plasmid-mediated *mcr-1* gene in Enterobacteriaceae is worrisome because this gene could facilitate the horizontal transfer of colistin resistance among different strains of bacterial species. If this transfer of colistin resistance were to occur, the resulting spread of infection would likely result in human infections caused by colistin-resistant Enterobacteriaceae.

In Thailand, all antibiotics have been prohibited for use as a growth promoter in food-producing animals by the Ministry of Agriculture and Cooperatives since 2015. Colistin has been prohibited for use to prevent infection in food-producing animals by the Department of Livestock Development, Ministry of Agriculture and Cooperatives since 2017. The aforementioned regulations are based on the observation that antibiotic-resistant bacteria on farms that used antibiotics as a growth promoter and colistin-resistant bacteria on farms that used colistin to prevent infection were much more prevalent than on farms that did not use antibiotics to promote growth and on farms that did not use colistin to prevent infection in food-producing animals, respectively. However, colistin is still permitted for use as the last option therapy for short-term treatment of infection in food-producing animals under the supervision of a qualified veterinarian.

The objective of the current study was to determine the occurrence of colistin-resistant Enterobacteriaceae in pigs raised on swine farms that received colistin for short-term treatment of infection, and the frequency of *mcr-1* in colistin-resistant Enterobacteriaceae isolated from pigs after receiving colistin for treatment of infection.

## Materials and methods

The Animal Care and Use Committee for Scientific Research of Kasetsart University (ACKU59-VET-002) approved this study. Formal approval was given by the owners of the 2 study farms for sample collection from study swine. This study was conducted at 2 large swine farms in Thailand and the microbiology laboratory of the Division of Infectious Diseases and Tropical Medicine, Department of Medicine, Faculty of Medicine Siriraj Hospital, Mahidol University, Bangkok, Thailand during October 2017 to April 2018.

### Swine farms

Two swine farms with more than 500 breeders each that are located in the Northeastern region of Thailand were included in this study. Neither of these two farms have used colistin to prevent infection for longer than one year. Halquinol and/or other antibiotics, such as ampicillin, tetracycline, tiamulin, and tilmicosin, was/were used to prophylactically prevent infection.

### Antibiotic treatment regimen for sick pigs on the 2 study swine farms

Sick pigs, especially pigs with post-weaning diarrhea, were treated with colistin sulphate 50,000 IU per pig via oral administration two times per day for up to 3 days. If a sick pig did not respond to colistin, other antibiotics, such as ampicillin, ceftriaxone, or enrofloxacin, would be given.

### Collection of rectal swab sample from pigs

Rectal swabs were collected from 12 pregnant sows on Farm A, and from 6 pregnant sows on Farm B within 30 days prior to parturition. Rectal swabs were also collected from 3 to 5 pigs born to each sow at birth, and on days 7, 14, 21, 28, and 60. Additional rectal swab samples were collected from sick pigs that were treated with colistin. All rectal swabs taken were maintained in Cary Blair transport medium and subjected to bacterial culture, antibiotic susceptibility testing, and *mcr-1* gene detection in colistin-resistant Enterobacteriaceae at the Division of Infectious Diseases and Tropical Medicine, Department of Medicine, Faculty of Medicine Siriraj Hospital, Mahidol University.

### Management of rectal swab sample collected from pigs

Each rectal swab sample was streaked on MacConkey agar supplemented with ceftriaxone 4 mg/L. The bacteria grown on the agar were identified up to the species level for Enterobacteriaceae (*Escherichia coli*, *Klebsiella pneumoniae*, *Enterobacter* spp., *Citrobacter freundii*, and *Edwardsiella tarda*) by conventional biochemical testing. Antibiotic susceptibility of the isolated Enterobacteriaceae to ceftriaxone and meropenem was performed by disk diffusion test according to the Clinical and Laboratory Standards Institute (CLSI) [20]. Determination of extended-spectrum beta-lactamase (ESBL)-producing Enterobacteriaceae was performed by double disk synergy test using a ceftriaxone disk (30 μg), a ceftazidime disk (30 μg), and an amoxicillin/clavulanic acid disk (2:1 30 μg). Colistin resistance of Enterobacteriaceae was screened by colistin disk (10 μg). All of the antibiotic disks used in this study were purchased from Oxoid, Basingstoke, England. Enterobacteriaceae isolates that exhibited a colistin inhibition zone diameter ≤11 mm were withheld for further determination of colistin minimum inhibitory concentration (MIC) by broth microdilution method according to CLSI [20]. Enterobacteriaceae isolates with a colistin MIC ≥4 mg/L were considered resistant to colistin. Determination of the presence of the *mcr-1* gene in colistin-resistant Enterobacteriaceae isolates was performed by polymerase chain reaction (PCR) amplification using the commercial primer with the same sequence as the *mcr-1* gene, as previously described by the researchers who discovered the *mcr-1* gene [18].

### Data analysis

SPSS Statistics (SPSS, Inc., Chicago, IL, USA) was used to perform all statistical analyses. Data specific to antibiotic resistance, the presence of ESBL in isolated Enterobacteriaceae, and the presence of the *mcr-1* gene in colistin-resistant Enterobacteriaceae isolates were described as number and percentage. Comparison of the prevalence of the *mcr-1* gene in Enterobacteriaceae isolates was performed using chi-square statistics or Fisher’s exact test. A *p*-value less than or equal to 0.05 was considered statistically significant

## Results

### Enterobacteriaceae isolates from sows and their antibiotic resistance

Among the Enterobacteriaceae isolates that were detected from 18 pregnant sows within 30 days before parturition, most (70%) were *E*. *coli*, and some were *K*. *pneumoniae* or *Citrobacter freundii*. The prevalence of resistance to ceftriaxone, meropenem, or colistin; of ESBL production; and, of the presence of the *mcr-1* gene in Enterobacteriaceae isolated from rectal swabs collected from 2 pregnant sows on Farm A, and from 6 pregnant sows on Farm B within 30 days before parturition is shown in Table 1. At least one Enterobacteriaceae isolate from all sows on both farms was resistant to ceftriaxone and was an ESBL producer. None of the Enterobacteriaceae isolated from pregnant sows on either farm was resistant to meropenem or colistin within 30 days before parturition.

**Table 1:** Prevalence of resistance to ceftriaxone, meropenem, or colistin; of ESBL production; and, of the presence of the *mcr-1* gene in Enterobacteriaceae isolated from pregnant sows.

Pregnant sows within 30 days before parturition		
Antibiotic resistance and presence of ESBL and *mcr-1* gene	Farm A (N = 12)	Farm B (N = 6)
Ceftriaxone-resistant Enterobacteriaceae	12 (100%)	6 (100%)
ESBL-producing Enterobacteriaceae	12 (100%)	6 (100%)
Meropenem-resistant Enterobacteriaceae	0 (0.0%)	0 (0.0%)
Colistin-resistant Enterobacteriaceae	0 (0.0%)	0 (0.0%)
Presence of *mcr-1* gene in colistin-resistant Enterobacteriaceae	N/A	N/A

**Abbreviations:** ESBL, extended spectrum beta-lactamase; *mcr-1* gene, mobilized plasmid-borne colistin resistance gene; N/A, not applicable.

### Enterobacteriaceae isolates from pigs born to study sows and their antibiotic resistance

**Fig 1:**
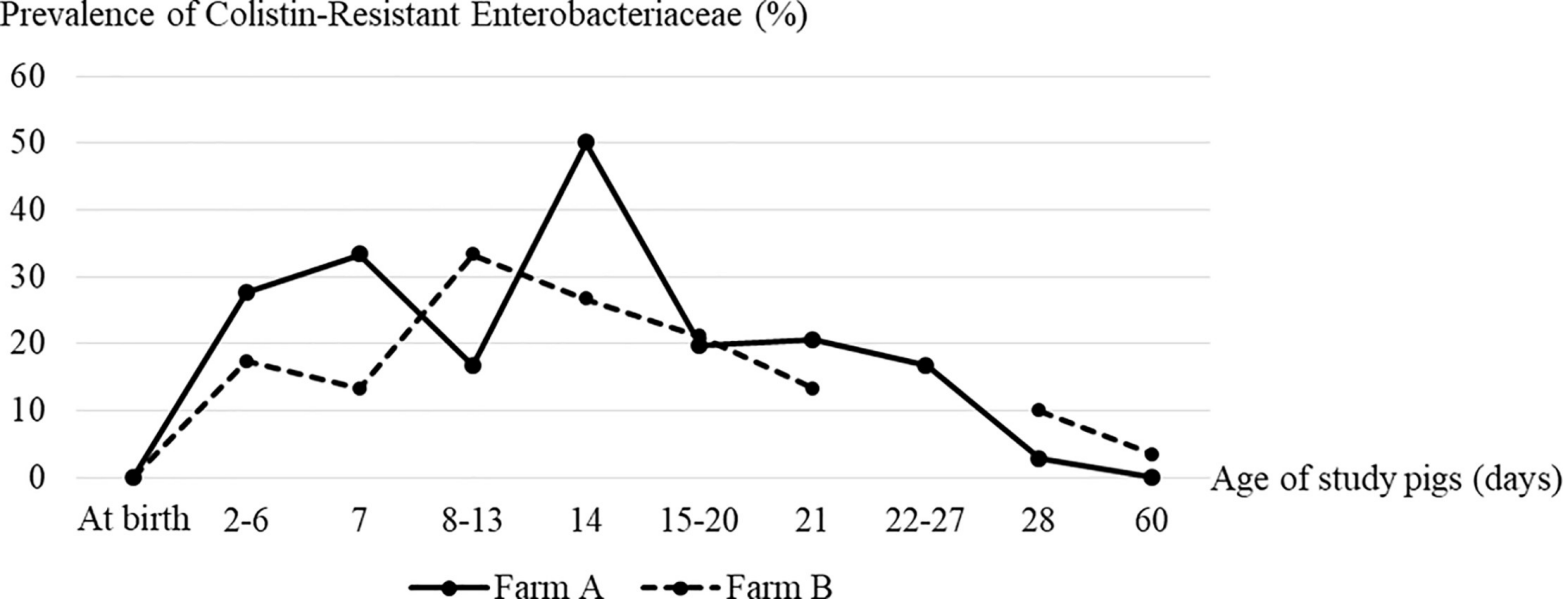
Prevalence of colistin-resistant Enterobacteriaceae isolates from study pigs from birth to age 60 days on Farm A and Farm B.

None of the study pigs born to study sows were sick at birth. However, 25 pigs (69.4%) on Farm A had diarrhea and received colistin treatment during days 2–27 after birth, and 23 (76.7%) on Farm B had diarrhea and received colistin treatment during days 3–21 after birth. Among the Enterobacteriaceae isolates detected from all study pigs from birth to day 60 after birth most (54.1% to 100%) were *E*. *coli*, and some were *K*. *pneumoniae*, *Enterobacter* spp., *Citrobacter freundii*, or *Edwardsiella tarda*. Table 2 shows the prevalence of resistance to ceftriaxone, meropenem, or colistin; of ESBL production; and, of the presence of the *mcr-1* gene in Enterobacteriaceae isolated from pigs born to study sows on Farm A and Farm B from birth to day 60 after birth. At least one Enterobacteriaceae isolate from all study pigs from birth to day 60 after birth on both farms was resistant to ceftriaxone in 91.2% to 100%. At least one Enterobacteriaceae isolate from all study pigs from birth to day 60 after birth on both farms was an ESBL-producer in 86.1% to 100%. None of the Enterobacteriaceae isolates from any of the study pigs on either farm was resistant to meropenem. Colistin-resistant Enterobacteriaceae isolates were not detected in any study pig at birth on either farm. At least one colistin-resistant Enterobacteriaceae isolate from study pigs aged 2 days to 27 days on Farm A (during which 69.4% of pigs had diarrhea and received colistin treatment) was detected in 16.7% to 50%. At least one colistin-resistant Enterobacteriaceae isolate from study pigs aged 3 days to 21 days on Farm B (during which 76.7% of pigs had diarrhea and received colistin treatment) was detected in 13.3% to 33%. Colistin-resistant Enterobacteriaceae were isolated from one pig (2.8%) aged 28 days on Farm A, and from 3 pigs (10%) aged 28 days on Farm B when all study pigs on both farms were not sick and they were not receiving colistin. Colistin-resistant Enterobacteriaceae were isolated from no pigs aged 60 days on Farm A, and from one pig (3.4%) aged 60 days on Farm B when all study pigs on both farms were not sick and they were not receiving colistin. Fig 1 shows the prevalence of colistin-resistant Enterobacteriaceae isolates from study pigs from birth to age 60 days on Farm A and Farm B. Colistin resistance emerged in Enterobacteriaceae isolates collected by the rectal swabs of pigs after colistin was used to treat sick pigs on Farm A and Farm B. The prevalence of colistin-resistant Enterobacteriaceae isolates from study pigs was highest in pigs aged 8 to 14 days on both farms, and it was very low or absent after colistin was discontinued.

**Table 2:** Prevalence of resistance to ceftriaxone, meropenem, or colistin; of ESBL production; and, of the presence of the *mcr-1* gene in Enterobacteriaceae isolated from pigs born to study sows.

**Healthy pigs at birth**		
**Antibiotic resistance and presence of ESBL and *mcr-1* gene**	**Farm A (N = 36)**	**Farm B (N = 30)**
Ceftriaxone-resistant Enterobacteriaceae	33 (91.2%)	30 (100%)
ESBL-producing Enterobacteriaceae	31 (86.1%)	30 (100%)
Meropenem-resistant Enterobacteriaceae	0 (0.0%)	0 (0.0%)
Colistin-resistant Enterobacteriaceae	0 (0.0%)	0 (0.0%)
Presence of *mcr-1* gene in colistin-resistant Enterobacteriaceae	N/A	N/A
**Sick pigs aged 2 to 6 days**		
**Antibiotic resistance and presence of ESBL and *mcr-1* gene**	**Farm A (N = 29)**	**Farm B (N = 23)**
Ceftriaxone-resistant Enterobacteriaceae	29 (100%)	23 (100%)
ESBL-producing Enterobacteriaceae	29 (100%)	23 (100%)
Meropenem-resistant Enterobacteriaceae	0 (0.0%)	0 (0.0%)
Colistin-resistant Enterobacteriaceae	8 (27.6%)	4 (17.4%)
Presence of *mcr-1* gene in colistin-resistant Enterobacteriaceae	5 (62.5%)	3 (75.0%)
**Healthy and sick pigs aged 7 days**		
**Antibiotic resistance and presence of ESBL and *mcr-1* gene**	**Farm A (N = 36)**	**Farm B (N = 30)**
Ceftriaxone-resistant Enterobacteriaceae	36 (100%)	29 (96.7%)
ESBL-producing Enterobacteriaceae	36 (100%)	28 (93.3%)
Meropenem-resistant Enterobacteriaceae	0 (0.0%)	0 (0.0%)
Colistin-resistant Enterobacteriaceae	12 (33.3%)	4 (13.3%)
Presence of *mcr-1* gene in colistin-resistant Enterobacteriaceae	6 (50.0%)	3 (75.0%)
**Sick pigs aged 8 to 13 days**		
**Antibiotic resistance and presence of ESBL and *mcr-1* gene**	**Farm A (N = 30)**	**Farm B (N = 54)**
Ceftriaxone-resistant Enterobacteriaceae	30 (100%)	53 (98.1%)
ESBL-producing Enterobacteriaceae	30 (100%)	53 (98.1%)
Meropenem-resistant Enterobacteriaceae	0 (0.0%)	0 (0.0%)
Colistin-resistant Enterobacteriaceae	5 (16.7%)	18 (33.3%)
Presence of *mcr-1* gene in colistin-resistant Enterobacteriaceae	0 (0.0%)	15 (83.3%)
**Healthy and sick pigs aged 14 days**		
**Antibiotic resistance and presence of ESBL and *mcr-1* gene**	**Farm A (N = 35)**	**Farm B (N = 30)**
Ceftriaxone-resistant Enterobacteriaceae	34 (97.1%)	30 (100%)
ESBL-producing Enterobacteriaceae	35 (100%)	29 (96.7%)
Meropenem-resistant Enterobacteriaceae	0 (0.0%)	0 (0.0%)
Colistin-resistant Enterobacteriaceae	17 (50.0%)	8 (26.7%)
Presence of *mcr-1* gene in colistin-resistant Enterobacteriaceae	6 (35.3%)	7 (87.5%)
**Sick pigs aged 15 to 20 days**		
**Antibiotic resistance and presence of ESBL and *mcr-1* gene**	**Farm A (N = 66)**	**Farm B (N = 57)**
Ceftriaxone-resistant Enterobacteriaceae	61 (92.4%)	57 (100%)
ESBL-producing Enterobacteriaceae	65 (98.5%)	55 (96.5%)
Meropenem-resistant Enterobacteriaceae	0 (0.0%)	0 (0.0%)
Colistin-resistant Enterobacteriaceae	13 (19.7%)	12 (21.1%)
Presence of *mcr-1* gene in colistin-resistant Enterobacteriaceae	4 (30.8%)	10 (83.3%)
**Healthy and sick pigs aged 21 days**		
**Antibiotic resistance and presence of ESBL and *mcr-1* gene**	**Farm A (N = 34)**	**Farm B (N = 30)**
Ceftriaxone-resistant Enterobacteriaceae	32 (94.1%)	30 (100%)
ESBL-producing Enterobacteriaceae	34 (100%)	29 (96.7%)
Meropenem-resistant Enterobacteriaceae	0 (0.0%)	0 (0.0%)
Colistin-resistant Enterobacteriaceae	7 (20.6%)	4 (13.3%)
Presence of *mcr-1* gene in colistin-resistant Enterobacteriaceae	4 (57.1%)	4 (100%)
**Sick pigs aged 22 to 27 days**		
**Antibiotic resistance and presence of ESBL and *mcr-1* gene**	**Farm A (N = 6)**	**Farm B (N = 0)**
Ceftriaxone-resistant Enterobacteriaceae	6 (100%)	N/A
ESBL-producing Enterobacteriaceae	6 (100%)	N/A
Meropenem-resistant Enterobacteriaceae	0 (0.0%)	N/A
Colistin-resistant Enterobacteriaceae	1 (16.7%)	N/A
Presence of *mcr-1* gene in colistin-resistant Enterobacteriaceae	1 (100%)	N/A
**Healthy pigs aged 28 days**		
**Antibiotic resistance and presence of ESBL and *mcr-1* gene**	**Farm A (N = 35)**	**Farm B (N = 30)**
Ceftriaxone-resistant Enterobacteriaceae	35 (100%)	30 (100%)
ESBL-producing Enterobacteriaceae	32 (91.4%)	30 (100%)
Meropenem-resistant Enterobacteriaceae	0 (0.0%)	0 (0.0%)
Colistin-resistant Enterobacteriaceae	1 (2.8%)	3 (10.0%)
Presence of *mcr-1* gene in colistin-resistant Enterobacteriaceae	0 (0.0%)	2 (66.7%)
**Healthy pigs aged 60 days**		
**Antibiotic resistance and presence of ESBL and *mcr-1* gene**	**Farm A (N = 35)**	**Farm B (N = 29)**
Ceftriaxone-resistant Enterobacteriaceae	35 (100%)	29 (100%)
ESBL-producing Enterobacteriaceae	33 (94.2%)	29 (100%)
Meropenem-resistant Enterobacteriaceae	0 (0.0%)	0 (0.0%)
Colistin-resistant Enterobacteriaceae	0 (0.0%)	1 (3.4%)
Presence of *mcr-1* gene in colistin-resistant Enterobacteriaceae	N/A	0 (0.0%)

**Abbreviations:** ESBL, extended spectrum beta-lactamase; *mcr-1* gene, mobilized plasmid-borne colistin resistance gene; N/A, not applicable.

### Characteristics of colistin-resistant Enterobacteriaceae

Table 3 describes the characteristics of all colistin-resistant Enterobacteriaceae isolates from all study pigs on Farm A and Farm B. Among all colistin-resistant Enterobacteriaceae isolates, 79.5% and 13.9% were *E*. *coli* and *K*. *pneumoniae*, respectively. Nearly all isolates of colistin-resistant Enterobacteriaceae were ESBL-producers. The MIC50, MIC90, and MIC range of colistin for all colistin-resistant Enterobacteriaceae isolates was 8, >128, and 4 to >128 mg/L, respectively. The overall prevalence of *mcr-1* gene in colistin-resistant Enterobacteriaceae isolates was 57.6%. The prevalence of *mcr-1* gene in colistin-resistant *E*. *coli* isolates was significantly higher than the prevalence of *mcr-1* gene in colistin-resistant *K*. *pneumoniae* isolates (70.0% *vs*. 9.5%, respectively; *p*<0.01).

**Table 3:** Characteristics of all isolates of colistin-resistant Enterobacteriaceae from all study pigs.

Colistin-resistant Enterobacteriaceae	n	ESBL-producer	Colistin MIC50/MIC90 (MIC range)	*mcr-1* positive
*E*. *coli*	120 (79.5%)	119 (99.2%)	8/>128 (4 to >128 mg/L)	84 (70.0%)
*K*. *pneumoniae*	21 (13.9%)	21 (100%)	16/64 (4 to >128 mg/L)	2 (9.5%)
*Enterobacter* spp.	5 (3.3%)	5 (100%)	8/16 (4 to >128 mg/L)	0 (0.0%)
*Citrobacter freundii*	4 (2.6%)	4 (100%)	8/8 (8 to >128 mg/L)	0 (0.0%)
*Edwardsiella tarda*	1 (0.7%)	1 (100%)	N/A	1 (100%)
All	151 (100%)	150 (99.3%)	8/>128 (4 to >128 mg/L)	87 (57.6%)

**Abbreviations:** ESBL, extended spectrum beta-lactamase; MIC, minimum inhibitory concentration; *mcr-1* gene, mobilized plasmid-borne colistin resistance gene; *E*. *coli*; *Escherichia coli*; *K*. *pneumoniae*, *Klebsiella pneumoniae*; N/A, not applicable.

## Discussion

This study was conducted on two large swine farms in Thailand that had not used colistin to prevent infection for at least one year to ensure that the outcomes of the study would not be adversely influenced by its recent use. However, these farms still used other antibiotics, including ampicillin, tetracyclines, tiamulin, tilmicosin, and/or halquinol, to prevent infection in their respective swine populations. Rectal swabs from pregnant sows and study piglets at birth revealed no isolates of colistin-resistant Enterobacteriaceae. Therefore, 13.3% to 50.0% of sick and healthy pigs with colistin-resistant Enterobacteriaceae isolated from their rectal swabs were associated with colistin use for treatment of sick pigs during day 2 to day 27. The prevalence of colistin-resistant Enterobacteriaceae isolated from pigs during the period that colistin was used to treat sick pigs in this study was higher than the previously reported prevalence [6], but it was comparable to the 47.5% prevalence of colistin-resistant Enterobacteriaceae isolated from hospitalized patients who were receiving colistin [5]. The emergence of colistin resistance in Enterobacteriaceae isolated from pigs in this study within a few days after colistin treatment developed very rapidly. This finding is consistent with the reported 3 days that it took for drug resistance to emerge in an experiment in swine, and the reported 7 days it took in patients receiving colistin treatment for infections [5, 21]. Marked decrease or disappearance of colistin-resistant Enterobacteriaceae in pigs after cessation of colistin use has also been reported [5, 21, 22]. The observed rapid emergence of colistin resistance in Enterobacteriaceae after exposure to colistin, and the rapid disappearance of colistin resistance in Enterobacteriaceae after cessation of colistin use are very interesting, and our group is investigating the mechanisms of this phenomenon. We are planning an *in vitro* study that will expose colistin to colistin-susceptible Enterobacteriaceae and determine when and how often the study colistin-susceptible Enterobacteriaceae become resistant to colistin. Then, we will grow colistin-resistant Enterobacteriaceae in media without colistin for many rounds to determine when and how often the study colistin-resistant Enterobacteriaceae will revert to colistin-susceptible Enterobacteriaceae. The colistin-susceptible Enterobacteriaceae and colistin-resistant Enterobacteriaceae isolates will be studied by whole genome sequencing to determine the relevant mechanisms of this phenomenon. The presence of the *mcr-1* gene was far more prevalent in colistin-resistant *E*. *coli* than in colistin-resistant *K*. *pneumoniae* isolated from pigs in this study. This finding is similar to the previously reported detection rate of the *mcr-1* gene in colistin-resistant *E*. *coli* (84.6%) and colistin-resistant *K*. *pneumoniae* (1.9%) isolated from patients [23]. We do not know the reason why the *mcr-1* gene was found to be more prevalent in *E*. *coli* than in *K*. *pneumoniae* in this study, and in the study in humans. We are collaborating with several genome centers to perform whole genome sequencing of colistin-resistant *E*. *coli* and *K*. *pneumoniae*. Those investigations may reveal additional mechanisms of colistin resistance in *E*. *coli* and *K*. *pneumoniae* other than *mcr-1* that could be mediated by other new plasmids [24, 25], chromosomes (e.g., PmrAB, PhoPQ, MgrB, and/or PmrD), and/or new mechanisms.

Although colistin resistance can disappear from Enterobacteriaceae after cessation of colistin, the short-term use of colistin is still worrisome since it poses several risks related to the emergence, transfer, and transmission of colistin-resistant bacteria. Colistin sulfate is poorly absorbed through a pig’s gastrointestinal tract [21]. Thus, colistin can potentially induce bacteria residing in the gastrointestinal tract of pig to become resistant to colistin, which could be mediated by resistant plasmid *mcr-1*. However, other resistant genes in plasmid (e.g., *mcr-2-7*) and chromosomal-mediated colistin resistance could also be a mechanism of colistin resistance in bacteria residing in the gastrointestinal tract of pigs that are exposed to colistin. Unabsorbed colistin in a pig is excreted from the pig’s gastrointestinal tract into the environment, and this could induce colistin resistance among bacteria that reside in the environment by various colistin resistance mechanisms similar to the bacteria in the gastrointestinal tracts of pigs that received colistin. Colistin-resistant Enterobacteriaceae that emerge in pigs receiving colistin can be transmitted to other pigs that do not receive colistin, and this would contaminate pork that is consumed by human beings, and potentially contaminate the environment [26–30]. Moreover, the plasmid-mediated *mcr-1* gene could potentially be transferred horizontally from colistin-resistant Enterobacteriaceae to other species of bacteria and become resistant to colistin [6]. Therefore, a risk-benefit analysis should be conducted before recommending colistin for treatment of infection in food animals.

It should be noted that Enterobacteriaceae isolated from all pigs in this study were generally resistant to ceftriaxone and were ESBL-producers because many antibiotics in addition to colistin were still given to the pigs on these two farms to prevent and treat bacterial infections. Fortunately, no carbapenem-resistant Enterobacteriaceae (CRE) were detected among all of the samples obtained from pigs in this study. Our finding is in contrast to previous studies that reported the isolation of CRE from pigs. CRE is one of the most important antibiotic-resistant bacteria linked to high mortality in human beings [31, 32].

Although this study clearly demonstrates association between the use of colistin for short-term treatment of infections and the emergence of colistin-resistant Enterobacteriaceae in swine, our study also has some mentionable limitations. First, our study data was collected from only 2 farms, and both of those farms are located in the same region of Thailand; therefore, the magnitude of the prevalence of colistin-resistant Enterobacteriaceae in swine observed from this study may not be generalizable to other farms since the data obtained from these 2 study farms were also different between farms. Second, the prevalence of colistin-resistant Enterobacteriaceae in swine observed from this study should be considered as minimum prevalence because we used MacConkey agar supplemented with ceftriaxone 4 mg/L for culture of the samples collected from pigs, and some colistin-resistant isolates of Enterobacteriaceae might be missed if such isolates were susceptible to ceftriaxone. However, the aforementioned phenomenon is unlikely since colistin resistance was not detected in any ceftriaxone-susceptible Enterobacteriaceae in our laboratory. Third, we did not attempt to detect variants of *mcr-1* and other types of *mcr* genes since we do not have the primers for these genes. Therefore, the prevalence of colistin resistance mediated by *mcr* genes in colistin-resistant Enterobacteriaceae isolates in this study could be more than the prevalence of the *mcr-1* gene alone that was observed in this study.

## Conclusion

Short-term treatment of sick pigs with colistin was found to be associated with emergence of colistin-resistant Enterobacteriaceae in swine raised on two large swine farms in Thailand. Colistin-resistant Enterobacteriaceae rapidly emerged after colistin administration, and colistin-resistant Enterobacteriaceae rapidly decreased or disappeared after colistin use was discontinued.

## Supporting information

S1 File (DOC)
