# Supplementary material for: Association between the use of colistin for short-term treatment of Gram-negative bacterial infections and the emergence of colistin-resistant Enterobacteriaceae in swine from selected swine farms in Thailand
Source: PLoS One. 2020 Oct 5;15(10):e0238939. doi: 10.1371/journal.pone.0238939 (PMC7535051; doi:10.1371/journal.pone.0238939)
Supplement: S1 File — (DOC) [file pone.0238939.s001.doc]

Minimum inhibitory concentration (MIC) of colistin per strain of isolated Enterobacteriaceae

| Organism | MIC of Colistin (mg/L) |
| --- | --- |
| Escherichia coli | 4 |
| Escherichia coli | 4 |
| Escherichia coli | 4 |
| Escherichia coli | 4 |
| Escherichia coli | 4 |
| Escherichia coli | 4 |
| Escherichia coli | 4 |
| Escherichia coli | 4 |
| Escherichia coli | 4 |
| Escherichia coli | 4 |
| Escherichia coli | 4 |
| Escherichia coli | 4 |
| Escherichia coli | 4 |
| Escherichia coli | 4 |
| Escherichia coli | 4 |
| Escherichia coli | 4 |
| Escherichia coli | 4 |
| Escherichia coli | 4 |
| Escherichia coli | 4 |
| Escherichia coli | 4 |
| Escherichia coli | 4 |
| Escherichia coli | 4 |
| Escherichia coli | 4 |
| Escherichia coli | 4 |
| Escherichia coli | 4 |
| Escherichia coli | 4 |
| Escherichia coli | 4 |
| Escherichia coli | 4 |
| Escherichia coli | 4 |
| Escherichia coli | 4 |
| Escherichia coli | 4 |
| Escherichia coli | 4 |
| Escherichia coli | 4 |
| Escherichia coli | 4 |
| Escherichia coli | 4 |
| Escherichia coli | 4 |
| Escherichia coli | 4 |
| Escherichia coli | 4 |
| Escherichia coli | 4 |
| Escherichia coli | 4 |
| Escherichia coli | 4 |
| Escherichia coli | 4 |
| Escherichia coli | 4 |
| Escherichia coli | 4 |
| Escherichia coli | 4 |
| Escherichia coli | 4 |
| Escherichia coli | 4 |
| Escherichia coli | 4 |
| Escherichia coli | 4 |
| Escherichia coli | 4 |
| Escherichia coli | 4 |
| Escherichia coli | 4 |
| Escherichia coli | 8 |
| Escherichia coli | 8 |
| Escherichia coli | 8 |
| Escherichia coli | 8 |
| Escherichia coli | 8 |
| Escherichia coli | 8 |
| Escherichia coli | 8 |
| Escherichia coli | 8 |
| Escherichia coli | 8 |
| Escherichia coli | 8 |
| Escherichia coli | 8 |
| Escherichia coli | 8 |
| Escherichia coli | 8 |
| Escherichia coli | 8 |
| Escherichia coli | 8 |
| Escherichia coli | 8 |
| Escherichia coli | 8 |
| Escherichia coli | 8 |
| Escherichia coli | 8 |
| Escherichia coli | 8 |
| Escherichia coli | 8 |
| Escherichia coli | 8 |
| Escherichia coli | 8 |
| Escherichia coli | 8 |
| Escherichia coli | 8 |
| Escherichia coli | 8 |
| Escherichia coli | 8 |
| Escherichia coli | 8 |
| Escherichia coli | 8 |
| Escherichia coli | 8 |
| Escherichia coli | 8 |
| Escherichia coli | 8 |
| Escherichia coli | 8 |
| Escherichia coli | 8 |
| Escherichia coli | 8 |
| Escherichia coli | 8 |
| Escherichia coli | 8 |
| Escherichia coli | 8 |
| Escherichia coli | 8 |
| Escherichia coli | 8 |
| Escherichia coli | 8 |
| Escherichia coli | 8 |
| Escherichia coli | 8 |
| Escherichia coli | 8 |
| Escherichia coli | 8 |
| Escherichia coli | 8 |
| Escherichia coli | 8 |
| Escherichia coli | 8 |
| Escherichia coli | 8 |
| Escherichia coli | 8 |
| Escherichia coli | 16 |
| Escherichia coli | 16 |
| Escherichia coli | >128 |
| Escherichia coli | >128 |
| Escherichia coli | >128 |
| Escherichia coli | >128 |
| Escherichia coli | >128 |
| Escherichia coli | >128 |
| Escherichia coli | >128 |
| Escherichia coli | >128 |
| Escherichia coli | >128 |
| Escherichia coli | >128 |
| Escherichia coli | >128 |
| Escherichia coli | >128 |
| Escherichia coli | >128 |
| Escherichia coli | >128 |
| Escherichia coli | >128 |
| Escherichia coli | >128 |
| Klebsiella pneumoniae | 4 |
| Klebsiella pneumoniae | 4 |
| Klebsiella pneumoniae | 8 |
| Klebsiella pneumoniae | 8 |
| Klebsiella pneumoniae | 8 |
| Klebsiella pneumoniae | 8 |
| Klebsiella pneumoniae | 8 |
| Klebsiella pneumoniae | 16 |
| Klebsiella pneumoniae | 16 |
| Klebsiella pneumoniae | 16 |
| Klebsiella pneumoniae | 16 |
| Klebsiella pneumoniae | 16 |
| Klebsiella pneumoniae | 16 |
| Klebsiella pneumoniae | 16 |
| Klebsiella pneumoniae | 16 |
| Klebsiella pneumoniae | 16 |
| Klebsiella pneumoniae | 32 |
| Klebsiella pneumoniae | 32 |
| Klebsiella pneumoniae | 64 |
| Klebsiella pneumoniae | 64 |
| Klebsiella pneumoniae | >128 |
| Enterobacter spp. | 4 |
| Enterobacter spp. | 8 |
| Enterobacter spp. | 8 |
| Enterobacter spp. | 16 |
| Enterobacter spp. | >128 |
| Citrobacter freundii | 8 |
| Citrobacter freundii | 8 |
| Citrobacter freundii | 8 |
| Citrobacter freundii | >128 |
| Edwardsiella tarda | 8 |
